# Sensory encoding and memory in the mushroom body: signals, noise, and variability

**DOI:** 10.1101/lm.053825.123

**Published:** 2024-05

**Authors:** Moshe Parnas, Julia E. Manoim, Andrew C. Lin

**Affiliations:** 1 Department of Physiology and Pharmacology, Faculty of Medicine, Tel Aviv University, Tel Aviv 69978, Israel; 2 Sagol School of Neuroscience, Tel Aviv University, Tel Aviv 69978, Israel; 3 School of Biosciences, University of Sheffield, Sheffield S10 2TN, United Kingdom; 4 Neuroscience Institute, University of Sheffield, Sheffield S10 2TN, United Kingdom

## Abstract

To survive in changing environments, animals need to learn to associate specific sensory stimuli with positive or negative valence. How do they form stimulus-specific memories to distinguish between positively/negatively associated stimuli and other irrelevant stimuli? Solving this task is one of the functions of the mushroom body, the associative memory center in insect brains. Here we summarize recent work on sensory encoding and memory in the *Drosophila* mushroom body, highlighting general principles such as pattern separation, sparse coding, noise and variability, coincidence detection, and spatially localized neuromodulation, and placing the mushroom body in comparative perspective with mammalian memory systems.

When you learn to associate a particular stimulus with a positive or negative experience, how does your brain ensure that the association is specific to that particular stimulus and not other similar but irrelevant stimuli? For example, a child touching a hot stove in the kitchen may learn to associate the hot stove with pain and therefore to avoid it. However, the same child will most probably not avoid a toy stove located in their room. What mechanisms allow this associative memory to be stimulus-specific?

Studies in mammalian brains have provided us with many mechanistic insights (Burgess and O'Keefe 2011; Kandel et al. 2014; Eichenbaum 2017; Mel et al. 2017; Fernández and Morris 2018; Josselyn and Tonegawa 2020). However, the sheer number of neurons and neuronal types and the overall complexity of the mammalian memory circuitry make it difficult to fully understand the underlying mechanisms.

The *Drosophila* olfactory system is an ideal model to study the mechanisms underlying stimulus-specific associative memory. The *Drosophila* nervous system consists of only ∼10^5^ neurons, but it shares many information-coding principles with more complex mammalian brains. Furthermore, recent technical advances have allowed the complete ultrastructural connectome of the fly nervous system to be reconstructed, revealing novel connections and neuron types (Galili et al. 2022). Finally, the *Drosophila* model system has numerous genetic tools that allow neurons to be labeled and manipulated at single-cell resolution, whether for electrophysiological recording, functional imaging, altering gene expression, or activating/silencing identified neurons in behaving animals (Venken et al. 2011). Thus, *Drosophila* allows us to simplify the complexity of the mammalian brain by stripping it down to its most fundamental building blocks and dissecting the underlying mechanisms in a simple, genetically accessible nervous system.

In this review, we address the mechanisms underlying stimulus-specific associative memory in the fly's memory center, the mushroom body, with a particular focus on pattern separation between sensory inputs, mitigation and exploitation of noise and variability, and the specificity of neuromodulation.

## Olfactory memory in *Drosophila*

In classical conditioning, animals learn to associate a conditioned stimulus (CS), such as odor, taste, touch, etc., with an unconditioned stimulus (US) that signals punishment or reward. In *Drosophila* olfactory learning, flies learn to associate specific odors (CS) with electric shock punishment or sugar reward (US). We begin by describing the early olfactory system, which carries the CS odor information to the fly's memory center.

**Figure 1.**
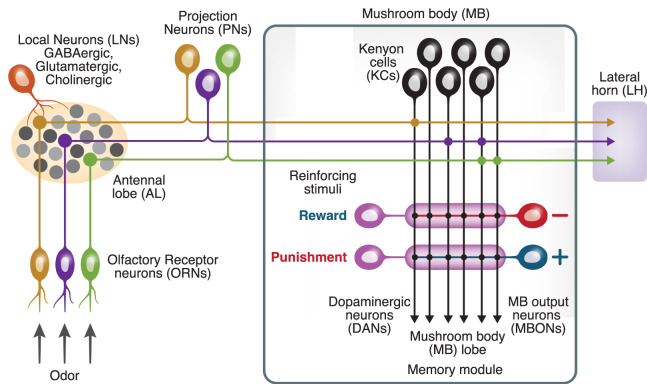
Diagram of the *Drosophila* olfactory system. Odors activate olfactory receptor neurons (ORNs). ORNs signal to matching projection neurons (PNs; indicated by matching colors: brown, purple, and green) via glomeruli in the antennal lobe; these signals are transformed by local neurons (LNs). PNs project to the mushroom body for flexible behavior and to the lateral horn for innate behavior. In the mushroom body, PNs activate Kenyon cells (KCs), which respond sparsely to odors. Each KC sends axons through different compartments. In compartments for appetitive memory, KCs get local neuromodulatory input from reward-encoding dopaminergic neurons (DANs) and send output to mushroom body output neurons (MBONs) that trigger avoidance behavior (–). Conversely, in compartments for aversive memory, KCs get input from punishment-encoding DANs and send output to MBONs that trigger approach behavior (+). Learning occurs by depressing outputs to the “wrong” action.

The organization of the insect and mammalian olfactory systems shows striking similarities. Odors are sensed by olfactory receptor neurons (ORNs), with each ORN expressing only one type of olfactory receptor in most cases (Couto et al. 2005; Fishilevich and Vosshall 2005; Benton et al. 2009). ORNs project to the antennal lobe, the first relay of the olfactory circuit, with all ORNs that express the same receptor converging onto the same glomerulus (Grabe et al. 2016). In *Drosophila*, there are 51 olfactory glomeruli (Bates et al. 2020). Second-order projection neurons (PNs) send their dendrites to AL glomeruli; while these are morphologically and neurochemically diverse (Tanaka et al. 2012; Bates et al. 2020), of greatest interest for learning are the uniglomerular cholinergic PNs, each of which sends dendrites to a single glomerulus and thereby receives monosynaptic input from only a single class of ORNs. PNs, in turn, project onto two brain regions: the mushroom body (MB), which underlies olfactory learning and memory, and the lateral horn (LH), which is involved in odor intensity and odor valence coding (Parnas et al. 2013; Dolan et al. 2019; Frechter et al. 2019; Lerner et al. 2020). The principal neurons of the MB, called Kenyon cells (KCs), are where the CS (odor) and the US (reward/punishment) converge (Fig. 1).

Whereas the CS is signaled by odor-evoked KC activity, the US is signaled by the activity of dopaminergic neurons (DANs). Different DANs signal punishment or reward (Das et al. 2014; Lin et al. 2014b; Cohn et al. 2015; Huetteroth et al. 2015; Dylla et al. 2017; Berry et al. 2018; Villar et al. 2022) and innervate spatially segregated compartments along the KC axon bundles (Fig. 1; Tanaka et al. 2008; Aso et al. 2014a; Li et al. 2020; Otto et al. 2020). These compartments match the innervation patterns of mushroom body output neurons (MBONs): Each MBON innervates a specific compartment along the KC axon bundles (Tanaka et al. 2008; Aso et al. 2014a). These MBONs are excited by KCs and can be considered as valence-encoding neurons, as their optogenetic activation leads to either attraction or avoidance (Aso et al. 2014b; Owald et al. 2015). The compartments pair DANs with MBONs of the “opposite” valence: Reward DANs innervate the same compartment as MBONs that drive aversion, and punishment DANs innervate the same compartment as MBONs that drive attraction (Aso et al. 2014a,b).

This anatomical arrangement suggests that learning occurs by weakening the “incorrect” action. Indeed, when an odor is paired with, say, reward, the coincidence of KC activity and “reward” DAN activity weakens synapses from the odor-activated KCs onto “avoidance” MBONs; conversely, KC activity plus “punishment” DAN activity weakens outputs toward “approach” MBONs (Séjourné et al. 2011; Hige et al. 2015; Owald et al. 2015; Perisse et al. 2016; Berry et al. 2018; Felsenberg et al. 2018; Stahl et al. 2022; Modi et al. 2023; Yamada et al. 2023). This synaptic depression is specific to the compartment innervated by the activated DAN; it does not spread to neighboring compartments (Hige et al. 2015; Stahl et al. 2022). Thus, learned behavior is thought to be driven by a plasticity-induced imbalance between avoid and approach MBONs.

Ultrastructural studies suggest that dopamine is broadcast globally within each compartment (Takemura et al. 2017). Thus, all KCs in each compartment, not just odor-activated KCs, receive the signal to weaken their synaptic outputs to that compartment's MBON. How, then, are olfactory memories kept odor-specific? According to current models, one key factor is that plasticity is restricted to KCs that were activated by the trained odor because the plasticity requires coincident activity of KCs and DANs. In this way, other KCs would not undergo depression at their output synapses, so untrained odors would activate only synapses untouched by training. This principle in turn creates two further requirements. First, odor specificity requires Kenyon cells to encode the CS in a way that allows efficient pattern separation. Second, neuromodulation should be effective on a KC only when dopamine arrives at the same time that the KC is active. We now turn to the mechanisms underlying these requirements.

## Pattern separation by sparse odor coding in Kenyon cells

**Figure 2.**
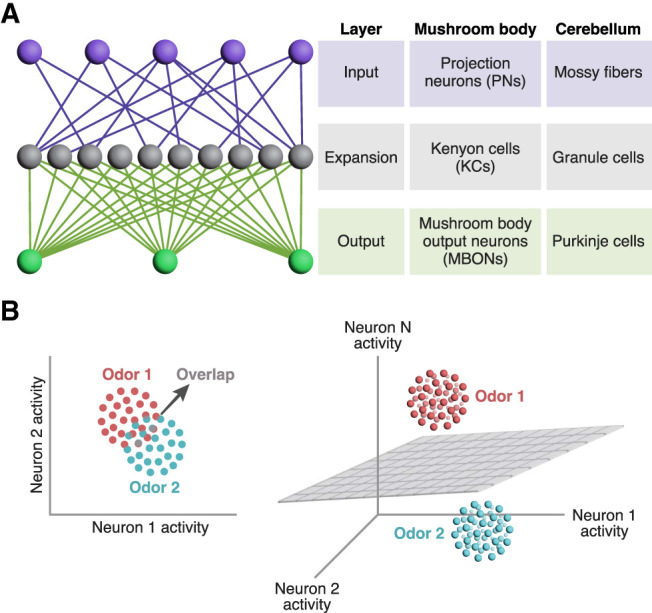
Expansion recoding aids pattern separation. (*A*) Diagram of expansion recoding circuitry. In expansion layer circuits, a small number of input layer neurons (purple) synapse onto a much larger number of expansion layer neurons (gray), which in turn converge onto a small number of output layer neurons (green). This basic architecture is used by both the insect mushroom body and the vertebrate cerebellum. (*B*) Why expansion recoding aids discrimination. Each dot represents the neuronal response to a single exposure to an odor in “activity space,” where each dimension is one neuron's activity. Due to noise, different trials elicit slightly different responses, represented by the cluster of dots of the same color. Here, in a two-dimensional space, the clusters for odor 1 and odor 2 overlap, making it difficult to cleanly discriminate between the two odors by drawing a line between the two clusters. In contrast, in three-dimensional space, the two clusters can be separated by a plane. The same principle applies if the red dots are rewarded odors and the blue dots are punished odors. Adapted from Cayco-Gajic and Silver (2019).

The mushroom body is one example among many in the animal kingdom of so-called “expansion layer” circuits, in which a relatively small number of input channels (projection neurons) project onto a much larger number of “expansion layer” neurons (Kenyon cells). The expansion layer then converges on a small number of output neurons (MBONs), and learning occurs by modifying synapses from the expansion layer to the output layer (Fig. 2A). This expand–converge structure (sometimes called a “fan-out, fan-in” structure) was noted decades ago, including in the mushroom body issue of *Learning & Memory*, for which this issue marks the 25th anniversary (Heisenberg 1998; Laurent et al. 1998). This basic architecture is also found in the cerebellum, the electrosensory lobe of weakly electric fish, and the hippocampus (Warren and Sawtell 2016; Cayco-Gajic and Silver 2019; Modi et al. 2020). It is also widely used in machine learning, where it is known by various names such as perceptrons, reservoir computing, echo state networks, or extreme learning machines (here, the expansion layer is generally called a “hidden” layer) (Rosenblatt 1958; Huang et al. 2006; Tanaka et al. 2019). Theoreticians in the 1960s and 1970s suggested that this expansion layer design allows for “expansion recoding”—transforming dense codes in the input layer, where a large fraction of neurons out of a small number are active, into sparse codes in the expansion layer, where only a small fraction of neurons out of a large number are active (Marr 1969; Albus 1971; Kawato et al. 2021).

Expansion recoding has at least two benefits. First, it can increase the dimensionality of sensory representations (Litwin-Kumar et al. 2017); that is, the number of dimensions needed to describe population responses to different sensory stimuli in “activity space.” High dimensionality is important for associative learning because it enables stimuli to be classified by linear separators; i.e., lines and planes (Cayco-Gajic and Silver 2019). To understand this intuitively, one may imagine red and blue dots mixed together on a two-dimensional plane; because they are mixed together, they cannot be separated by a straight line. However, in three-dimensional space, the red and blue dots might no longer be mixed, so they could be separated by a plane (i.e., “linearly separable”) (Fig. 2B). A similar principle applies to “hyperplanes” in higher-dimensional spaces. This linear separability—the ability to classify stimuli by separating them using hyperplanes—is important because the learning algorithm (modifying the output synapses of expansion layer neurons) is equivalent to drawing a hyperplane in activity space to divide stimuli. Thus, high-dimensional odor representations in Kenyon cells allow synaptic plasticity at their output synapses to separate odors into rewarded and punished classes.

Second, expansion recoding allows sparse responses (Olshausen and Field 2004). The large number of expansion layer neurons allows each neuron to respond to only a small fraction of stimuli (“lifetime” sparseness) and allows each stimulus to activate only a small fraction of neurons (“population” sparseness). Compared with projection neurons, KC responses are sparser in both lifetime and population sparseness; ∼5%–10% of KCs respond reliably to each odor presentation (although ∼20% respond unreliably) (see below; Perez-Orive et al. 2002; Turner et al. 2008; Honegger et al. 2011). For example, in one set of experiments, odors activated on average 59% of PNs but only 6% of KCs (Turner et al. 2008). This sparseness aids odor specificity of memory because only KCs activated by the CS^+^ (the odor paired with the US) depress their output synapses onto MBONs. If a new odor that was never paired with reward/punishment activates a CS^+^-activated KC, this new odor would activate previously depressed KC–MBON synapses and thereby inappropriately trigger a learned behavior. Thus, sparse KC odor responses reduce overlap between odor representations and thereby make olfactory memories more specific.

This model is supported by both experimental and computational results. When KC odor responses are made less sparse by removing feedback inhibition from the GABAergic interneuron APL (anterior paired lateral), flies are less able to learn to discriminate between similar odors (Lin et al. 2014a). Moreover, flies are less able to discriminate between pairs of odors or odor mixtures whose KC representations are more overlapping (Campbell et al. 2013; Hige et al. 2015; Ahmed et al. 2023; Modi et al. 2023). Finally, computational studies show improved memory performance with sparser activity in the expansion layer (Babadi and Sompolinsky 2014). Indeed, under some circumstances, lifetime sparseness of Kenyon cells is a better predictor of memory performance than dimensionality (Abdelrahman et al. 2021).

If sparseness aids learned discrimination, why not use a maximally sparse code, where only a single KC responds to each odor? Such a “grandmother cell” strategy (so called from the tongue-in-cheek idea that one's grandmother is represented by a single neuron in the visual cortex) (Barwich 2019) would only be able to encode as many odors as there are KCs (2000) and would not be robust to noise: If an odor activates only a single KC, any failure of this KC to respond (due to intertrial variation in KC activity) (Honegger et al. 2011; Srinivasan et al. 2023) would make the flies fail to detect the odor.

Moreover, discrimination is not the sole function of the mushroom body: In order to recognize different encounters with one odor as the same odor, the flies must generalize between noisy KC representations of the same odor (Fig. 2B). As most odor objects are complex mixtures of chemicals, in nature it is likely also useful to generalize between similar odor mixtures. Here, overlap between KC representations is helpful rather than harmful.

Thus, sparse—but not extremely sparse—KC coding may strike a balance between discrimination and generalization/robustness. Computational studies have shown that the sparseness of expansion layer representations controls a trade-off between discrimination and generalization (Barak et al. 2013) and that expansion layer responses are more variable between trials when they are extremely sparse (<10% of cells responding) (Litwin-Kumar et al. 2017). Encoding odors in sparse subsets rather than single cells may also allow the mushroom body to exploit intertrial variability to aid discrimination: Whereas the core of reliable cells that always respond to an odor is helpful for distinguishing very different odors, these cells may be too overlapping between very similar odors to allow discrimination. Instead, the “penumbra” of unreliable cells that only sometimes respond to an odor may be more helpful in discriminating between very similar odors (Srinivasan et al. 2023).

Moreover, the interodor overlap in KC representations carries useful information about odor similarity. Odors eliciting more similar ORN activity patterns also elicit more similar KC activity patterns, and the relative similarity of different odor pairs is stereotyped across individuals (Turner et al. 2008; Lin et al. 2014a; Endo et al. 2020; Yang et al. 2023). In addition, while PN–KC connectivity is mostly random, some PN channels are overrepresented or underrepresented as KC inputs (Caron et al. 2013; Hayashi et al. 2022; Ellis et al. 2023), and certain food-related PN channels are more likely than chance to coinnervate the same KCs (Zheng et al. 2022). This random but biased input to KCs is predicted to make certain ethologically relevant odors more salient for learning and make it easier to generalize between odors from similar natural sources (Zavitz et al. 2021; Ellis et al. 2023; Yang et al. 2023). Indeed, across insects, mice, and humans, similarity in neuronal odor representations matches not physico–chemical similarity between odor molecules but rather how close the molecules are in “metabolic space” (i.e., how few metabolic steps it takes to transform one molecule into another in biological systems) (Qian et al. 2023). By encoding ethologically relevant odor similarity as overlap in KC odor representations, the mushroom body can generalize where appropriate. For example, an MBON known as MBONα′3 responds to novel odors but not familiar odors, yet novel odors that are similar to familiar odors are treated as partially familiar to the extent of the similarity, in a biological implementation of a data structure for testing similarity called a Bloom filter (Hattori et al. 2017; Dasgupta et al. 2018, 2022).

Indeed, whether flies discriminate or generalize between similar odors depends on behavioral context. *Drosophila* larvae discriminate between similar odors if, during training, one was presented with a reward and the other was presented without, but they generalize if only one odor was presented during training (Mishra et al. 2010). In adults, if A is paired with optogenetic punishment (artificial activation of punishment-encoding dopaminergic neurons) but similar odor A′ is not, flies discriminate between A and A′ when given a direct choice between them but generalize the aversive memory from A to A′ if given the choice between A′ and unrelated odor B, or if they experience A and A′ separated by a gap in time. This difference arises from the fact that when choosing between A and A′, flies experience transitions between the two odors (Modi et al. 2023). This phenomenon may arise from the temporal dynamics of learning: When KC activity precedes dopamine, KC output synapses are depressed, but when dopamine precedes KC activity, KC output synapses are potentiated (see “Stimulus-Specific Neuromodulation” below; Handler et al. 2019). Meanwhile, some KCs respond to odor onset and others respond to odor offset (Ito et al. 2008; Lüdke et al. 2018). Thus, when an odor is paired with punishment, “on” KCs’ outputs to approach MBONs are depressed because their activity preceded dopamine, while “off” KCs’ outputs are potentiated because their activity followed dopamine (Vrontou et al. 2021). This differential depression/potentiation means that if A is punished and A′ is not, then an approach MBON will fire more on transitioning from A to A′ than vice versa, leading the flies to prefer A′ even though they would avoid both A and A′ compared with B (Modi et al. 2023).

## Development and homeostasis of sparse coding

How is sparse, distributed coding established in Kenyon cells? In part, KCs are odor-specific because their relatively high spiking thresholds mean a KC requires multiple PN inputs to be simultaneously active to trigger it to fire an action potential (Gruntman and Turner 2013; Li et al. 2013). In addition, the mostly random connectivity between PNs and KCs means that each KC receives inputs from a different subset of PN channels (Caron et al. 2013); thus, not only does a small fraction of KCs respond to each odor, but each odor elicits responses in different KCs. Finally, feedback inhibition from the GABAergic interneuron APL increases sparseness by suppressing KC activity (Lei et al. 2013; Lin et al. 2014a).

Theoretically, the more inputs a KC receives, the more likely it is to receive enough simultaneous inputs to fire. Indeed, increasing (decreasing) the number of PN inputs per KC increases (decreases) their likelihood of responding to an odor (Elkahlah et al. 2020; Ahmed et al. 2023). Developmentally, the number of inputs per KC is set by KCs, not by PNs: When the number of PNs or KCs is artificially increased or decreased, PNs make more or fewer presynaptic boutons to meet the KCs’ altered “demand” for synapses, keeping the number of inputs per KC constant (Elkahlah et al. 2020; Puñal et al. 2021).

KC activity is also governed by homeostatic plasticity. When the inhibitory APL neuron is artificially activated for 4 d, the mushroom body adapts to the excess inhibition by increasing KC activity through a combination of decreased inhibition and increased excitation (Apostolopoulou and Lin 2020). This homeostatic compensation may also be useful for improving memory performance. Computational models show that natural variability in the parameters governing KC excitability (number/strength of excitatory inputs and spiking threshold) can impair memory performance because it increases variability in KC lifetime sparseness. This variability is a problem because some KCs respond very broadly and others do not respond at all, and these KCs are less useful for discriminating between odors (because they respond to too many odors or none at all). However, this problem can be solved by compensatory variability where, for example, KCs with higher spiking thresholds might have stronger excitatory inputs to compensate. Indeed, anatomical evidence for such compensation can be found in the hemibrain connectome (Scheffer et al. 2020), where, for example, KCs with more PN inputs have fewer synaptic sites per PN, suggesting weaker excitatory connections (Abdelrahman et al. 2021). Thus, homeostatic compensation may serve to mitigate the negative effects of interneuronal variability.

Homeostatic compensation has limits; for example, KCs do not compensate for complete loss of inhibition (Apostolopoulou and Lin 2020). Moreover, increasing the number of inputs per KC increases KC activity, making flies worse at discriminating similar odors (Ahmed et al. 2023), indicating that the KCs cannot fully compensate away the effect of increased excitation. Does this contradict the findings that KCs with more inputs have weaker inputs (Abdelrahman et al. 2021)? Not necessarily. First, perhaps the compensation mechanism itself requires adjusting of the input number, a process that would necessarily be bypassed when artificially altering the input number. Second, compensation in Kenyon cells might be imperfect: Kenyon cells with more inputs might weaken their excitatory inputs or raise their thresholds, but not by enough to completely erase the effect of more inputs.

## Stimulus-specific neuromodulation

Aside from efficient pattern separation by sparse KC odor coding, the second requirement for stimulus specificity is that dopamine should induce plasticity on KC output synapses only for KCs that are actually active when the dopamine arrives. This coincidence detection is enforced by multiple intracellular signaling mechanisms.

The first such mechanism is a pair of antagonistic signaling pathways thought to trigger depression versus potentiation of KC–MBON synapses. Triggering depression is a G_s_-coupled dopamine receptor (Dop1R1) that is expressed in KC presynaptic terminals and required for olfactory learning (Kim et al. 2007; Qin et al. 2012). Dop1R1 activates a downstream Ca^2+^-dependent adenylyl cyclase known as Rutabaga (Levin et al. 1992). It is believed that the coincidence of dopamine input with odor-evoked Ca^2+^ influx in KCs triggers cyclic adenosine monophosphate (cAMP)-dependent plasticity. Indeed, KC activation and dopamine application can synergistically elevate cAMP levels in KC axons in a Rutabaga-dependent manner (Tomchik and Davis 2009; Handler et al. 2019). This cAMP is thought to trigger KC–MBON depression to weaken the incorrect action (Fig. 1).

Conversely, flies that experience “reverse” pairing (US then CS) learn the opposite way: If flies experience electric shock followed by an odor, they learn to approach the odor because it predicts “relief” from pain (Tanimoto et al. 2004; Yarali et al. 2009; Aso and Rubin 2016; Handler et al. 2019; Jacob and Waddell 2020). This reverse plasticity also requires temporal proximity of KC activity and dopamine release. Here, the coincidence detector is thought to be the Ca^2+^-sensitive IP_3_ receptor, which likely induces stronger Ca^2+^ release from the ER in KCs when IP_3_ (triggered by the G_q_-coupled dopamine receptor Dop1R2) is followed by odor-evoked Ca^2+^ influx in KCs (Himmelreich et al. 2017; Handler et al. 2019), compared with the opposite order (Ca^2+^ then IP_3_). This Ca^2+^ release from the ER is thought to trigger potentiation of KC–MBON synapses to produce “reverse” learning (e.g., after shock then odor, approach outputs are potentiated because the odor predicts relief from pain).

**Figure 3.**
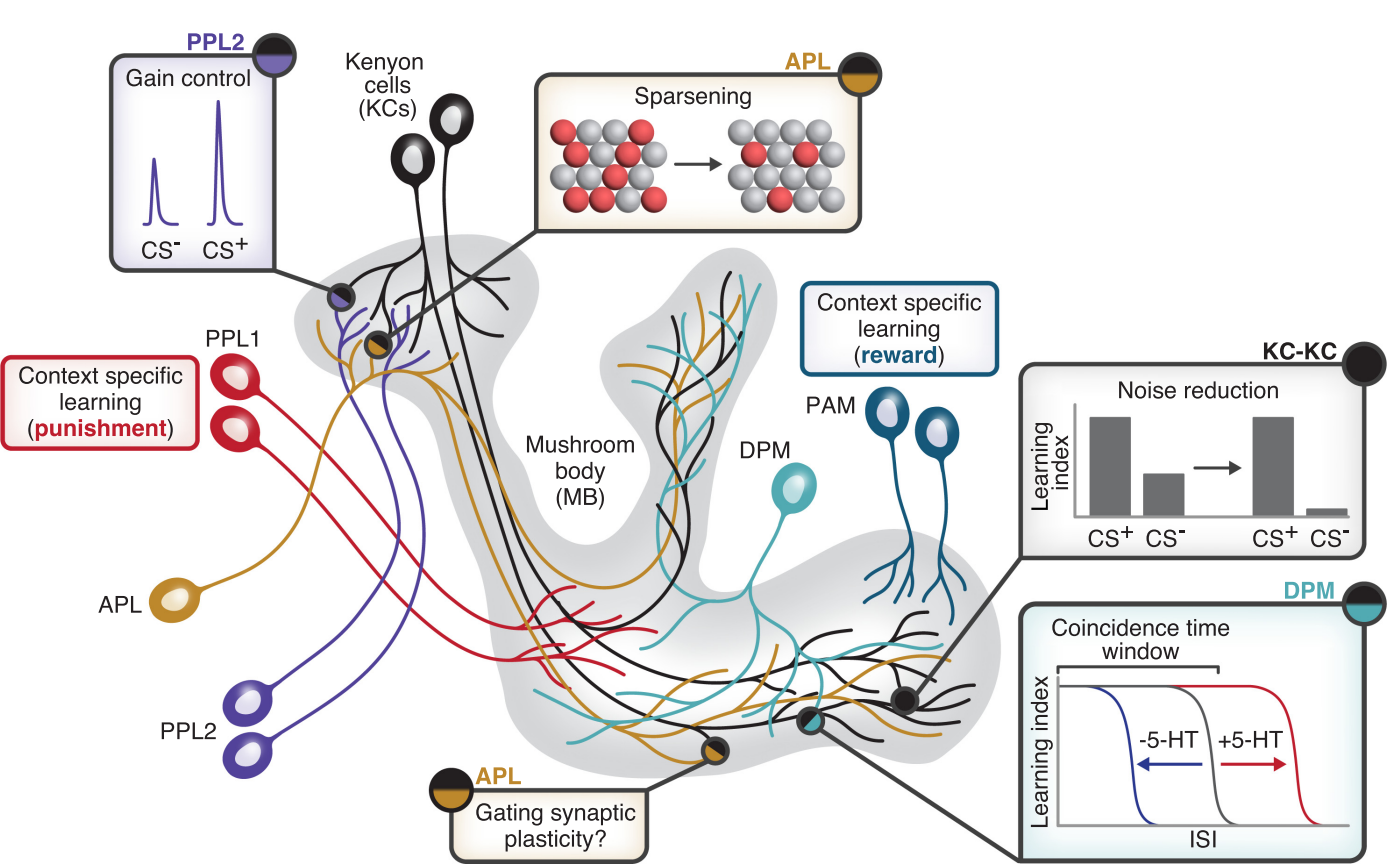
Local neuromodulation in the mushroom body. Illustration of examples of local neuromodulation. Dopaminergic PPL2 neurons (purple) innervate KC dendrites and enhance KC responses to the odor that was paired with the unconditioned stimulus (the CS^+^) but not the unpaired odor (the CS^−^). The GABAergic APL neuron (brown) innervates the whole mushroom body, but because it shows localized activity, it may have different functions on KC dendrites (sparsening KC odor responses) versus KC axons (perhaps gating synaptic plasticity). KC–KC synapses (black) implement axonal lateral inhibition via mAChR-B and thereby prevent flies from erroneously showing learned responses to the unpaired odor (CS^−^). The DPM neuron (turquoise) releases serotonin (5-HT) locally on KC axons and regulates the coincidence time window; i.e., the largest interstimulus interval (ISI; gap in time between the CS and US) that still allows flies to learn.

However, these coincidence detectors are not perfect. In particular, Rutabaga is still active in the absence of Ca^2+^ (Levin et al. 1992), so dopamine alone even without KC activity can increase cAMP levels (Tomchik and Davis 2009; Boto et al. 2014; Handler et al. 2019). One might expect this nonspecific cAMP elevation to depress the outputs even of KCs not activated by the trained odor, thus defeating the odor specificity allowed by sparse KC coding. The solution is twofold. First, cAMP production alone is not sufficient: A recent study showed that KCs require simultaneous cAMP production and KC depolarization in order to depress their presynaptic release probability (Yamada et al. 2024). Second, KCs form extensive axonal synapses with each other, as revealed in the connectome (Eichler et al. 2017; Takemura et al. 2017; Li et al. 2020). These lateral connections surprised the field at first because KCs are cholinergic (Barnstedt et al. 2016), and lateral excitation would, like nonspecific cAMP, defeat the benefit of the sparse coding in olfactory learning (Eichler et al. 2017). However, these lateral connections are actually inhibitory, mediated not by nicotinic receptors but by the inhibitory muscarinic receptor B (mAChR-B). mAChR-B is a GPCR coupled to G_i/o_ and reduces odor-evoked Ca^2+^ influx in KC axons (Manoim et al. 2022). Indeed, lateral inhibition via mAChR-B may explain why blocking the GABAergic feedback neuron APL has a smaller effect on KC activity than blocking synaptic output from all KCs (Lin et al. 2014a). Moreover, mAChR-B reduces cAMP synthesis, allowing lateral inhibition to prevent cAMP from increasing in the least active KCs, limiting synaptic depression to the most active KCs. Indeed, the loss of mAChR-B makes aversive olfactory memories less specific, so flies avoid even an untrained odor that has little overlap in KC representations with the trained odor (Fig. 3; Manoim et al. 2022).

Intriguingly, some dopaminergic neurons also corelease nitric oxide (NO), which triggers “inverted” learning (i.e., NO from reward DANs triggers aversive learning, and NO from punishment DANs triggers appetitive learning) at slower timescales (after ∼10 min), counteracting the dopamine-induced learning to promote forgetting (Aso et al. 2019). NO activates soluble guanylate cyclase (sGC), which produces cGMP and triggers transcriptional changes that promote forgetting (Takakura et al. 2023). sGC activity slowly potentiates KC–MBON synapses, but, as with cAMP, only when the KC is simultaneously active with sGC (Yamada et al. 2024).

## Compartmentalized neuromodulation locally modifies KC signaling

The axonal localization of mAChR-B highlights the compartmentalized signaling in KCs. As with many other neurons (Branco and Häusser 2010; Papoutsi et al. 2014), KCs cannot be understood simply as nodes in a neural network: They are complex three-dimensional structures with localized signaling in intracellular compartments. We described above how dopamine-triggered plasticity is restricted to particular axonal compartments, so that, for example, a punishment DAN weakens KC outputs to an approach MBON in the same compartment but not an avoidance MBON in a neighboring compartment (Fig. 1). As compartmentalized dopamine signaling on KCs has been well reviewed elsewhere (Cognigni et al. 2018; Hige 2018; Amin and Lin 2019; Felsenberg 2021; Siju et al. 2021), here we discuss recent examples of other types of local modulation of KC signaling.

### Dendritic gain control

Complementary to axonal mAChR-B, the muscarinic receptor mAChR-A is expressed exclusively in KC dendrites. Although mAChR-A is normally G_q_-coupled and thus usually excites neurons (Rozenfeld et al. 2019), in KCs, mAChR-A (like mAChR-B) is actually inhibitory (Bielopolski et al. 2019). mAChR-A signaling may reflect feed-forward inhibition from the cholinergic PNs or lateral inhibition between KCs: As with KC axons, KC dendrites also contain KC–KC synapses (Christiansen et al. 2011; Amin et al. 2020; Scheffer et al. 2020). mAChR-A is required for both olfactory learning and learning-induced depression of KC–MBON synapses (Silva et al. 2015; Bielopolski et al. 2019). It remains unclear exactly how dendritic mAChR-A signaling regulates axonal synaptic plasticity. We suggest two ideas. First, mAChR-A might improve pattern separation through lateral inhibition, similarly to mAChR-B. Although knocking down mAChR-A did not affect interodor correlations in KC activity at their cell bodies (Bielopolski et al. 2019), such measurements may have missed subtle effects or those restricted to KC dendrites/axons. It will be interesting to test whether the loss of mAChR-A reduces the specificity of olfactory memories. Second, mAChR-A might regulate synaptic plasticity “competence” rather than being involved in the plasticity mechanism itself. That is, mAChR-A activation may make flies’ learning mechanisms more sensitive but only when the flies smell some odors (i.e., cholinergic input arriving at KCs).

Another mechanism that enhances learning sensitivity is dopamine release in the calyx from so-called PPL2 neurons. Unlike the DANs in the mushroom body lobes, they do not themselves instruct associative memories; rather, their activity during CS^+^ + US pairing strengthens the activity of CS^+^-responsive KCs and makes memories stronger (Fig. 3; Boto et al. 2019). This memory “gain control” might occur because increased activity in CS^+^-responsive KCs enhances coincidence detection by Rutabaga or increases the salience of the CS^+^ odor. Indeed, long-term memory consolidation is correlated with structural and functional plasticity in PN–KC synaptic connections, including sharpened responses to the trained odor in KC dendrites (Baltruschat et al. 2021).

### Local inhibition

The feedback inhibitory neuron APL (see above; Fig. 3) is a nonspiking neuron (Papadopoulou et al. 2011) where activity does not readily spread far (estimated space constant ∼50 µm, compared with the ∼250-µm distance from the calyx to the tips of the axonal lobes) (Inada et al. 2017; Wang et al. 2019; Amin et al. 2020; Prisco et al. 2021). This spatially restricted spread means feedback inhibition in the mushroom body is localized even though APL innervates the entire mushroom body (Amin et al. 2020). Combined with the anatomical arrangement of KC–APL and APL–KC synapses, this result predicts that the median KC inhibits itself ∼40% more strongly than it inhibits other individual KCs (Amin et al. 2020). Local inhibition also suggests that the single-neuron APL may serve different functions on KC dendrites than on KC axons, essentially acting as multiple independent neurons (Grimes et al. 2010; Meier and Borst 2019). For example, on KC dendrites, it may primarily suppress KC spiking to enforce sparse coding, while on KC axons, it might locally gate KC–MBON plasticity (Fig. 3). Indeed, APL odor responses are suppressed by olfactory training, and this suppression enhances learning (Liu and Davis 2009; Zhou et al. 2019; Okray et al. 2023). It is tempting to speculate that local gating of plasticity could provide a functional logic for why approach MBONs and avoid MBONs are spatially segregated from each other.

### Local serotonin

A similar kind of local gating occurs with another mushroom body interneuron, the serotonergic DPM (dorsal paired medial) neuron, which widely innervates all KC axons (but not their dendrites) (Waddell et al. 2000). While DPM has long been known to be involved in memory consolidation (Keene et al. 2004, 2006; Yu et al. 2005; Krashes et al. 2007; Krashes and Waddell 2008; Wu et al. 2011), recent studies suggest that it also locally modulates coincidence detection in KC axons. First, serotonin lengthens the coincidence detection window, which is the maximum interval between CS and US for which the animal still learns an association between the two (Fig. 3). Serotonin release from DPM is spatially heterogeneous across different axonal compartments, causing correspondingly different coincidence detection windows in the different compartments. Serotonin acts via G_i_-coupled 5HT-1A to reduce tonic cAMP (but not phasic cAMP) in KCs; this tonic cAMP suppression may help stimulus-evoked cAMP signals stand out better against the background so that even a widely separated CS and US can still depress KC–MBON synapses (Zeng et al. 2023).

Second, serotonin release from DPM also mediates multimodal binding (Okray et al. 2023). Flies can learn to associate a US with a multimodal CS (e.g., combined color–odor stimuli), and this learning enhances future recall of even single-modality stimuli (e.g., after learning to avoid banana + blue, flies later avoid blue alone more strongly than if they had been trained only to avoid blue alone). This memory enhancement occurs because the coincidence of dopamine with simultaneous activation of visual and olfactory KCs unlocks DPM acting as a compartment-specific excitatory bridge between visual and olfactory KCs, thus allowing cross-modal enhancement during memory recall. For example, for appetitive memories, DPM spreads activity between visual CS-responsive and olfactory CS-responsive KCs in the appetitive memory compartments but not the aversive memory compartments (Okray et al. 2023).

## The mushroom body in comparative perspective

While this review has focused on olfactory memory in *Drosophila*, the mushroom body underlies olfactory memory via similar mechanisms in other insects as well (Perez-Orive et al. 2002; Szyszka et al. 2005; Ito et al. 2008; Groh and Rössler 2020). Moreover, there are important parallels with other species and disciplines in the general principles that we have outlined: sparse coding, coincidence detection, and localized signaling. Sparse coding for pattern separation is used in other expansion layer systems like the cerebellum, hippocampus, and piriform cortex (Cayco-Gajic and Silver 2019; Modi et al. 2020; Endo and Kazama 2022). Indeed, the role of inhibition in maintaining sparse coding in KCs for learned discrimination in the mushroom body (Lin et al. 2014a) has been replicated in the architecturally analogous granule cells of the cerebellum (see Fig. 2A; Fleming et al. 2024). Moreover, the biological problem solved by the mushroom body (stimulus-specific associative memory) also appears in machine learning in a common problem called “catastrophic forgetting,” where newly learned information overwrites old information. Catastrophic forgetting can be alleviated by adopting features of the mushroom body that improve stimulus specificity, like sparse coding and compensatory variability to equalize KC average activity (Manneschi et al. 2023; Shen et al. 2023).

Coincidence detection between principal neurons (here, KCs) and neuromodulatory neurons (here, DANs) is also commonly seen in other learning systems. Famously, the coincidence of sensory input (CS) and serotonin (US) triggers associative learning in *Aplysia* using the same intracellular second messenger as the mushroom body: cAMP (Hawkins 1984). In mammals, in the primary reward substrate, the ventral tegmental area (VTA), it is believed that the coincidence of a weak glutamatergic input (CS) with a strong neuromodulatory cholinergic input (US) strengthens the glutamatergic input so that it alone can activate VTA dopaminergic neurons (Galaj and Ranaldi 2021). Similarly, in eyeblink conditioning, mammals learn to associate a CS (e.g., a tone) with an air puff to the eye (US; and thus learn to blink to the CS alone), because granule cells carrying the CS (like KCs) fire immediately before climbing fibers carrying the US (like DANs), and this coincidence depresses parallel fiber–Purkinje cell synapses (like KC–MBON synapses) (see Fig. 2A; Freeman and Steinmetz 2011). Remarkably, the order sensitivity of this coincidence dependence depends on IP_3_ receptors (Sarkisov and Wang 2008), just as in the mushroom body (Handler et al. 2019).

Extending classical conditioning to operant conditioning uses a similar but slightly more complex coincidence detection rule. For example, in the striatum, current models suggest that corticostriatal and thalamostriatal inputs representing the current sensory context (like KCs in this analogy) can depress or potentiate their synapses onto spiny projection neurons representing a potential action (like MBONs), depending on the presence or absence of a dopamine signal representing whether the chosen action led to a reward (like DANs) (Wickens et al. 2003; Frémaux and Gerstner 2016; Gerstner et al. 2018). This “three-way” coincidence detection (pre, post, and dopamine), also known as neuromodulated spike timing-dependent plasticity (STDP), differs slightly from the mushroom body, which detects coincidences only between presynaptic KCs and DANs (MBON activity is usually not required) (Hige et al. 2015; but see also Cassenaer and Laurent 2012; Pribbenow et al. 2022). Including the postsynaptic neuron allows the plasticity rule to implement operant conditioning (learning the consequences of one's own actions) as opposed to classical conditioning (learning associations between two stimuli). It will be interesting to see whether *Drosophila*’s mechanisms for enhancing the specificity of coincidence detection (e.g., mAChR-B) have an equivalent parallel in mammals.

What about localized signaling? The general principle of different dopaminergic inputs projecting to spatially distinct regions is conserved in the mammalian striatum (Watabe-Uchida and Uchida 2018). While it is unclear whether the striatum also compartmentalizes neuromodulation within neurons in the same way as the mushroom body does, the neuromodulated STDP described above is local in that STDP is restricted to the activated presynaptic and postsynaptic terminals (Brzosko et al. 2019). Moreover, it is increasingly recognized that understanding intracellular compartmentalization (e.g., in dendrites) is key to understanding neuronal signal processing (Branco and Häusser 2010; Papoutsi et al. 2014) and to relating natural and artificial learning algorithms (Richards and Lillicrap 2019).

Given these important parallels and the track record of the last 50 years (Quinn et al. 1974; Bellen et al. 2010), future research on the *Drosophila* mushroom body will likely continue to reveal fundamental insights into learning mechanisms with applications to both mammalian systems and artificial intelligence.
